# Prostate Cancer Treatment: Cryoablation in Context

**DOI:** 10.3390/cancers18132025

**Published:** 2026-06-23

**Authors:** Thomas Lilieholm, Michael C. Risk, David Jarrard, Erica Knavel Koepsel

**Affiliations:** 1 Department of Medical Physics, University of Wisconsin–Madison, Madison, WI 53705, USA; tlilieholm@wisc.edu; 2 Department of Urology, University of Wisconsin–Madison, Madison, WI 53705, USA; risk@urology.wisc.edu (M.C.R.); jarrard@urology.wisc.edu (D.J.); 3 Department of Interventional Radiology, University of Wisconsin–Madison, Madison, WI 53705, USA

**Keywords:** prostate, cryoablation, MRI, CT, ultrasound, salvage therapy, urology, focal therapy

## Abstract

One of the most commonly diagnosed malignancies in men worldwide, prostate cancer (PCa) is the subject of much study across various treatment approaches and medical specialties. Currently, 5-year survival rates exceed 90%, and research efforts have increasingly shifted towards approaches that improve quality of life, minimizing the likelihood and severity of morbidities associated with treatment. Focal cryoablation is an approach that has been steadily gaining traction in recent years, both for its reduced risk of morbidity and greater potential for salvage therapy, relative to standard treatment. The goal of this work is to discuss the safety and efficacy of focal cryoablation for prostate cancers and define it in context among other treatment approaches. Publications describing approaches to, results from, and the science behind PCa cryoablation were reviewed and collated, describing the current landscape. Additional comparisons were made against analogous approaches, including radiation, surgical resection, hormone therapy, and other ablation modalities. Currently available literature characterizes prostate cryoablation as an effective and well-tolerated approach for treating primary and recurrent PCa, although the data are limited by heterogeneous evidence, lack of standardized endpoints, and an absence of robust randomized comparisons. Cryoablation for prostate cancer continues to evolve, offering a minimally invasive treatment option for both primary and salvage prostate cancer patients, although additional long-term studies are needed.

## Simple Summary

As a particularly pervasive cancer, prostate cancer has garnered a well-developed treatment algorithm, which has led to particularly low mortality rates. Consequently, new advances in the domain tend to improve patient morbidities and procedural efficiency, leading to a diverse array of differing treatment approaches. Focal cryoablation is an emerging prostate cancer treatment approach associated with lower rates of morbidities compared to standard treatments due to its ability to ablate within strictly defined margins and native tissue-sparing capabilities. These same attributes make it favorable for salvage treatments, allowing re-treatments without damaging tissue more than necessary. Comparison of focal cryoablation against other approaches, in terms of morbidities and long-term outcomes, is warranted to inform future research directions and the decision-making process for clinicians and patients.

## 1. Introduction

Prostate cancer is the leading cancer diagnosis in men [1,2]. The American Cancer Society predicts approximately 300,000 new cases of prostate cancer and 36,000 deaths from prostate cancer will occur in 2026 [2]. One in eight men will be diagnosed with prostate cancer, with the average age of diagnosis being 68 years old [1,2]. Because of its prevalence, robust treatment algorithms have been created that rely primarily on radical prostatectomy, radiation therapy, hormonal therapy, or active surveillance. While these treatment modalities have demonstrated successful results at controlling disease, with patient 5-year survival rates around 90%, recurrence rates remain relatively high (up to 40% on long timelines), depending on risk group and treatment modality [3,4,5]. In addition, there are known morbidities associated with these standard of care therapies [6,7,8].

Interventional practice continues to gravitate towards minimally invasive procedures to minimize side effects. Trends show providers opting for laparoscopic cholecystectomy versus open cholecystectomy, if possible, or percutaneous ablation for liver tumors as opposed to hepatic resection [8,9]. The same evolution can be seen within the field of prostate care, affecting treatment algorithms for prostate cancer and benign prostatic hyperplasia (BPH). Cryoablation is one of our oldest ablative techniques, with the first generation cryoablation device developed in 1960 [10,11]. Since then, cryoablation has become a major player in the world of percutaneous ablation. It is commonly used for the treatment of renal malignancies, soft tissue masses, osseous lesions, lung lesions, and vascular malformations, among other pathologies [12]. Power output and cryoprobe placement provide fine control over the ablation zone, enabling it to be used in a variety of locations within the body and, due to the sub-freezing temperatures, render most cells non-viable as it reaches temperatures less than −40 °C [13].

Historically, cryoablation for prostate cancer has been performed in the operating room under ultrasound guidance, with many ablations characterized as whole gland ablations [14]. However, advanced imaging has changed accessibility to this region of the pelvis through a percutaneous approach [15,16]. A variety of imaging modalities have been used to guide percutaneous cryoablation of the prostate, including computed tomography (CT) and magnetic resonance imaging (MRI) in conjunction or without ultrasound guidance [14,15,17]. These advanced imaging tools offer increased tissue conspicuity and visualization of the treatment as it progresses with the aim of providing more targeted and accurate treatment approaches. They have also paved the way for non-whole gland therapies as they provide a robust monitoring tool following cryoablation to monitor for any recurrent, residual, or new disease.

Another increasingly relevant dynamic in modern medicine is patient autonomy. More attention has been given to providing care that is in line with the patient’s goals of treatment, potentially pertaining to side effects, outcomes, recovery preferences, or close contact experiences. This is particularly relevant for the prostate as prioritizing clinical endpoints; eliminating the malignancy can result in worsened functional outcomes such as erectile dysfunction and urinary incontinence. In this way, prostate cancer treatment represents a complex crossroads wherein clinicians and their patients must balance competing concerns, treating a relatively low-mortality disease while preserving an organ functionality that tends to be highly valued. Opting for focal therapy is often chosen to treat the malignancy while preserving functionality, but this bears the cost of an increased risk of recurrence. Clinicians are duty-bound to provide the best possible care for their patients. In this era of growing patient autonomy, that includes a responsibility to incorporate patients’ goals of care into their treatment plan.

This review will summarize current techniques for prostate cancer cryoablation, as well as current guidelines for treatment and variables therein, including intraoperative imaging selection, whole gland versus focal ablation, and primary versus salvage treatment, all in the context of other comparable treatment approaches. This will be supported by a brief overview of the recent literature reporting outcomes from cryoablation services. Although cryoablation is a particularly promising treatment approach, evaluation of outcomes is limited by heterogeneous evidence, lack of standardized endpoints, and an absence of robust randomized comparisons.

## 2. Current Guidelines in Prostate Cryoablation

The American Urological Association (AUA) and American Society for Radiation Oncology provide the current screening and treatment guidelines for prostate cancer [18]. The guidelines are broken into four main categories: early detection/screening, localized prostate cancer, advanced prostate cancer, and salvage therapy for prostate cancer. The early detection guidelines address using biomarkers (PSA), diagnostic imaging, and prostate biopsy for appropriate detection of prostate cancer. Risk stratification of patients should be based on their clinical T stage, serum PSA, Gleason Score (GS), and tumor volume. These four factors have been shown to align closely with the underlying aggressiveness of the disease, so as to also align the treatment with the disease severity and categorization as localized vs. advanced disease and subsequent appropriate algorithm.

The three main recommended therapies are active surveillance, radical prostatectomy with or without lymph node dissection, and radiation therapy with or without androgen deprivation therapy (ADT). Per the AUA, cryoablation may be recommended to patients with full disclosure of the limited high-quality evidence for this treatment. The category most strongly recommended for cryoablation is treatment of salvage prostate cancer after radiotherapy failure. Per the AUA recommendations, cryoablation for primary treatment should be offered in the setting of a clinical trial due to the lack of high-quality evidence.

The European Association of Urology’s recommendations are in alignment with the AUA and the European Society for Medical Oncology, only endorsing cryoablation as a salvage therapy. Intra-study variability in technique and small data sets limits the current literature on the topic and its incorporation into the guidelines more broadly. Prospective and randomized trials comparing cryoablation to standard of care therapies are being performed, but more are needed to fully demonstrate the potential of cryoablation [19].

Although comprehensive formal guidelines for focal cryoablation are still evolving, there are several criteria that have been published that help guide patient selection for current providers of prostate cryoablation [20]. It is generally accepted that the most favorable candidates for cryoablation are those patients with biopsy-proven, intermediate-risk prostate cancer (GS7, GG2-3), PSA < 20, lesion < 2 cm with disease that is focal or non-diffuse and confined to the prostate [21]. Genomics, comorbidities, and patient age are also important factors to include when considering candidacy for cryoablation. Favorable genomics, multiple comorbidities, and advanced age may tip the scale towards cryoablation. As with all our ablative techniques, patient selection is key to being able to apply the technologies within their limitations.

While standard of care therapies, surgery, and radiation are not without their risks. The most common adverse effects (AEs) of radical prostatectomy are urinary incontinence, erectile dysfunction, abscess, urethral stricture, bleeding or damage to the rectum or bowel, in addition to lymphocele, lymphedema, if a lymphadenectomy is performed [22]. The most common AE after radiation therapy are urinary retention, irritative bladder symptoms, bowel irritation, and bowel incontinence [7,8]. These AEs have the potential to significantly impact a patient’s quality of life.

With the availability of focal treatments for prostate cancer, there are also ongoing discussions regarding the need for radical therapies for all prostate cancers, given that a majority are low- to intermediate-risk [23]. By treating the dominant lesions, there is speculation that this lesion drives the biology of the disease and subsequent disease course, with secondary lesions often smaller and lower grade [24]. Prostate cancer is classically considered a multifocal disease, which has been cited as a critique against focal therapies. Additional sites of disease can be synchronous or metachronous, which could lead to incomplete treatment or multiple treatments during the patient’s disease course. Identifying which patients have the lowest risk of recurrence or advanced disease is critical to ensure we are using focal therapies appropriately. All patients undergoing focal therapy should have a systematic and targeted biopsy to ensure evaluation of the prostate for imaging-negative disease. Furthermore, patients who undergo focal therapies need to commit to close follow-up post-procedure to verify the treatment and monitor for in-field and out-of-field disease development. Programs that offer focal therapy should have a robust follow-up protocol established prior to treating patients to minimize losses to follow-up.

The leading biomarker for prostate cancer is prostate-specific antigen (PSA), which can be evaluated from a simple blood test [25]. Heightened blood serum levels are an indicator of the presence of malignancy, which is often confirmed with contrast-enhanced MRI, as well as a PSMA PET scan and subsequent biopsy. Biopsied tissue may be used to categorize the severity of the malignancy using guidelines such as the Gleason Score or D’Amico risk group [26,27]. In low-risk cases of PCa, active surveillance, intermittent PSA measurements, and biopsies to monitor and track the disease are often recommended [28]. Curative treatment follows for those with disease progression [28]. Intermediate to high-risk cases, typically categorized as a GS 7 or higher, call for more direct action. Cryoablation, in particular, is best suited to low- to intermediate -risk prostate cancers; higher risk characterizations typically call for more radical treatments such as prostatectomy or radiotherapy [28].

Following cryoablation, PSA levels are expected to drop significantly; this is generally considered a prognostic indicator of treatment efficacy [28]. Accordingly, serial PSA measurements are typically taken from patients in the months and years following treatment to monitor outcomes and detect any potential recurrences. However, there is no standard definition of BCR following focal therapy, which presents a challenge in the monitoring and follow-up of focal therapy patients. Most studies are using the Phoenix criteria (nadir + 2.0 ng/mL) or the ASTRO criteria (three consecutive PSA increases), but the validity of this is not well established in this population [28]. Heavy reliance on advanced imaging like contrast-enhanced MRI, PSMA PET, or Choline C-11 PET with or without biopsy is used in conjunction with PSA studies to monitor and verify residual or recurrence disease following cryoablation.

## 3. Prostate Cryoablation Techniques

### 3.1. Cryoablation Mechanism of Action

Cryoablation causes cell death through a combination of intracellular ice crystal formation, rupturing cell membranes as the tissue freezes, and osmosis due to fluid shifts during the freeze–thaw cycle, resulting in cell swelling and rupture as the ice melts [29]. In this way, both the freezing and thawing actions of cryoablation contribute to the destruction of malignant cells. To fully leverage this, it is recommended to perform cryoablations with multiple cycles of freezing and thawing to ensure sufficient cell death within an isotherm [30]. Freezing itself is achieved by leveraging the Joule–Thomson effect [29]. The needle-like cryoprobes contain chambers that allow controlled gas decompression within. Depending on the gas and its properties, this rapidly decreases or increases the local temperature, freezing or warming tissues adjacent to the cryoprobe, wherever it is positioned within the patient’s anatomy. While most gases cool with rapid expansion (argon, oxygen), others like helium and hydrogen increase their temperature with rapid expansion. Over the course of one procedure, several cryoprobes are placed into the prostate at once, and all are activated simultaneously. These cryoprobes each generate their own frozen volume, which will overlap and synergize with adjacent ones. Taken as a whole, this results in a deliberately sculpted isotherm. Notably, the visual isotherm, a signal void in MRI, does not correspond exactly to the ablating isotherm. Cell death occurs reliably at −40 °C, which is lower than the threshold at which freezing tissue creates a signal void in MRI [13]. In this way, the visual isotherm must extend slightly past the margins of the intended ablation zone for controlled treatment.

### 3.2. Focal Versus Whole Gland Cryoablation

**Figure 1:**
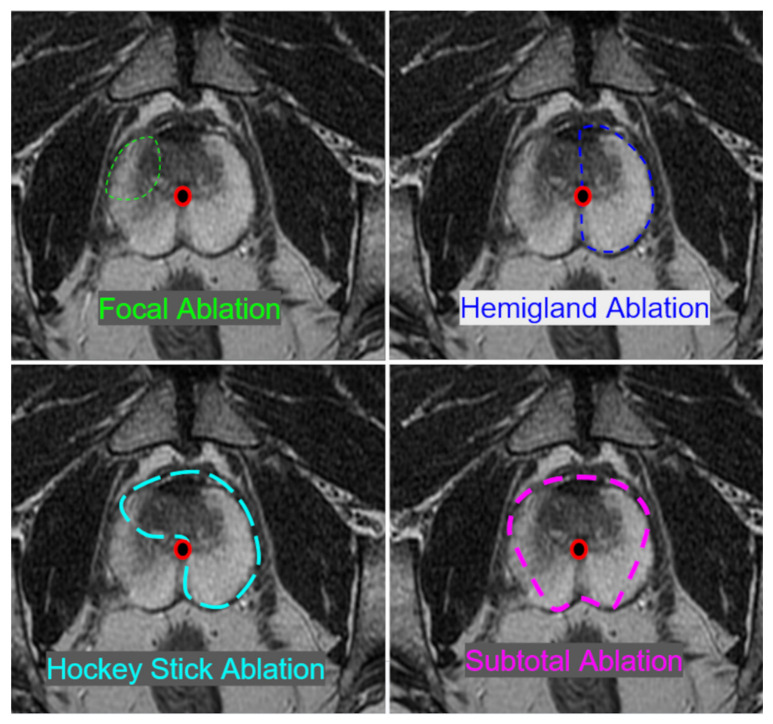
In anything less than whole gland cryoablation, subregions of the prostate are ablated to spare benign tissue. Care is always taken to avoid freezing the urethra (red), which passes through the center of the prostate. Small lesions are treated with focal ablation zones (green). Hemigland ablations (dark blue) target all tissue ipsilateral to the lesion. So-called hockey stick ablation patterns (cyan) extend this hemigland ablation zone across the midline while subtotal ablation (violet) targets most of the gland, sparing only the most lateral posterior regions.

Cryoablation as a technique has many parameters that may be adjusted to suit individual treatment cases on the basis of cancer staging and patient preference. Key among these is the distinction between focal and whole gland ablation paradigms. As with most cancer treatments, clinicians and patients must balance the tradeoffs between oncologic and functional outcomes. Whole gland cryoablation, naturally, freezes the entire prostate regardless of lesion location. While more thorough treatment as such tends towards superior oncologic outcomes, functional outcomes are often found to be worse, with increased rates of erectile dysfunction (ED) and urinary incontinence (UI) among patients, nearing the rates of prostatectomy or radiation, therefore negating the advantage of cryoablation. This trend appears in the data presented in this manuscript’s brief narrative literature review, though data availability is too limited to draw definitive conclusions. Ultimately, the decision to pursue focal or whole gland cryoablation is reliant on cancer staging and the patient’s priorities regarding functional outcomes. In general, cryoablation is recommended for cancers that are well-localized, non-diffuse and smaller in size (<2 cm) [28]; diffuse cancers, or cases with lesions throughout the prostate, or larger lesions may be better suited to more standard whole gland treatments [28]. This is reflected in the body of work reviewed here, wherein a majority of the cryoablation studies performed focal rather than whole-gland ablation. Typical ablation patterns include: hemi-gland ablation (anterior or posterior, right or left lateral prostate), hockey stick ablation (lateral ablation with extension across the midline, anteriorly or posteriorly), true focal ablation of the lesion alone or subtotal ablation, as shown in Figure 1 [31].

Advanced imaging techniques, like MRI, have opened the door to more focal therapies. Disease detection has improved with the sensitivity of MRI and PSMA PET, often allowing the detection of lesions earlier when they may be more amenable to focal therapy. Cryoablation performed using advanced imaging techniques also allows more accurate needle placement and the ability to monitor the ablation in near-real time to ensure coverage of the lesion and avoidance of structures like the rectum. Finally, post-ablation monitoring with regular PSA measurements (up to every 3 months for the first year), post-ablation imaging with contrast-enhanced MRI and PSMA PET, and potentially surveillance biopsy can detect treatment failures either in the field or out of the field, so additional treatment can be offered.

### 3.3. Primary Versus Salvage Cryoablation

Per societal guidelines, cryoablation for primary PCa treatment is recommended only for certain populations that are not candidates for radiation or surgery or if physicians are performing these ablations within the confines of a clinical trial. The lack of high-quality evidence supporting prostate cryoablation for primary cancer limits the more general use of this treatment option until more data can be acquired. The current standard of care paradigms for primary prostate cancer are supported by high-quality data. Nevertheless, biochemical recurrence rates for radical prostatectomy and radiation are reported at up to 40% and 57%, respectively, aggregated across risk groups and follow-up durations [32,33]. 

Salvage therapies can include salvage prostatectomy, salvage radiation (external beam or brachytherapy), androgen deprivation therapy, high-intensity focused ultrasound (HIFU) or cryoablation [18]. Salvage cryoablation is often a viable choice when there is a localized, non-metastatic recurrence, regardless of the primary treatment modality and provides a significant advantage for cryoablation [28]. Salvage cryoablation can be performed after radiation, prostatectomy, or prior ablation treatment failures, as well as in cases of pelvic or nodal recurrence. While repeat radiation treatments are often not recommended due to cumulative toxic effects of radiation in the tissues, increasing the risk of complications, cryoablation can be carefully performed following a radiation treatment failure, or even failure of a previous cryoablation treatment [18,28,34,35]. Additionally, cryoablation can be used to treat pelvic and lymph node recurrences post-surgery when re-operation is technically more challenging and prone to more complications [36]. Generally, salvage therapies are more difficult to draw conclusions from due to the variety of patients and cancer types included in these treatment arms, but the salvage patients represent a population that could benefit from an additional treatment option. The ablation zone can be carefully constructed and controlled and is well tolerated in previously treated tissues with an acceptable safety profile, preserving local collagenous structures [37]. This is an important distinction for cryoablation, allowing an effective, safe, and minimally invasive treatment in a setting where there are limited treatment options, mostly relying on chronic androgen deprivation therapy, which has a challenging side effect profile.

### 3.4. Intraoperative Imaging

As a minimally invasive and often focal treatment, PCa cryoablation is frequently paired with intraoperative imaging to enable greater safety and efficacy. The role of intraoperative imaging in this space is twofold. First, it enables accurate placement of cryoprobes into the tissue, which determines the ablation zone and ultimately, the success of the ablation. Inaccurately placed probes will fail to ablate the desired lesions and may even harm one or more of the several organs at risk (OARs) in the region. Intraoperative imaging allows clinicians to precisely plan their approach using cognitive fusion techniques and/or external trajectory guides [16] and, after the initial insertion, confirm accurate positioning with a second check scan. Second, intraoperative imaging techniques with sufficient time resolution (on the order of 10 s or faster) can be used to monitor the ablating iceball in real time. Since the frozen ablation zone expands radially out from the cryoprobes over time, visualization like this allows clinicians to verify that the actual ablation zone sufficiently covers the desired lesions while avoiding OARs. To ensure adequate cell death within the ablation zone, 2–3 cycles of freezing and thawing the same zone are typically preferred [30,38]. By repeatedly freezing and thawing the same tissue, greater cell death is achieved due to a combination of osmotic shock and ice crystal formation, rupturing cell membranes, leading to cell death. A single freeze cycle is not sufficient to guarantee complete cell death within the frozen tissue.

There are several different medical imaging modalities that may be employed in PCa cryoablation depending on the needs of the procedure and the particular strengths of each; sometimes, multiple modalities may be used in the same procedure. Key features that make an imaging method advantageous in PCa cryoablation are: clear anatomical visualization, clear delineation of the iceball, and real-time imaging capabilities. The predominant methods of intraoperative imaging used in prostate cryoablation are ultrasound, MRI, and CT. Multiple imaging modalities may be used simultaneously. Positron emission tomography (PET) imaging is often used in diagnostic planning images, but is not applied for intraoperative purposes.

Ultrasound is among the most widely used means of intraoperative image guidance due to its excellent real-time imaging capabilities in conjunction with its low cost, low risk, and convenience [39]. The prostate and adjacent tissues may be visualized via transrectal ultrasound (TRUS) or transperineal ultrasound, depending on the presence of other procedural hardware, the clinician’s preference, or simple availability of transducer models [40,41]. Ultrasound allows ready visualization of the prostate, inserted probes, and resultant iceballs with excellent temporal resolution for tracking the iceball. Though the ice–tissue interface in the near-field is readily apparent under ultrasound imaging, complete visualization of the ablation is not feasible as acoustic shadowing obscures the distal side or far-field of the iceball and any anatomy deep to this margin [42]. Only the edge of the iceball proximal to the transducer can be seen, limiting the clinician’s ability to fully assess the ablation zone. Further, ultrasound’s volumetric visualization is limited, with most transducers and displays limited to 2D viewplanes. This places additional uncertainty on the full 3-dimensional extent of the ablation zone, requiring clinicians to extrapolate volume from 2D images. Ultimately, ultrasound is the most readily available and convenient-to-use modality for intraoperative cryoablation imaging, but the information it conveys is inherently limited by the nature of the medium. Since ultrasound systems are portable, it is feasible to use ultrasound as a supplemental imaging approach for cases that rely more heavily on other modalities.

**Figure 2:**
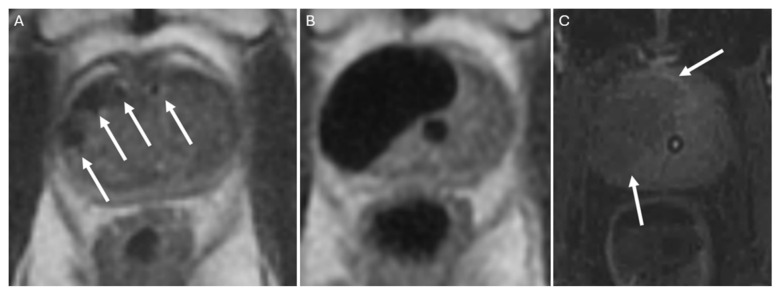
Inserting 4 cryoprobes ((**A**), white arrows) coaxially prescribes a volume of prostate tissue to be ablated when the probes are activated. Scans acquired during ablation clearly show this region (**B**) due to the signal void ice, which appears as in most sequences. Post-ablation scans, after the ice is thawed, can allow viewers to visualize and verify the extent of ablated tissue ((**C**), white arrows).

Magnetic resonance imaging occupies the far end of the spectrum from ultrasound. With superior soft tissue contrast and extensive multiparametric imaging capabilities, MRI offers the most detailed anatomical visualization. MR-derived signals clearly delineate the prostate and surrounding soft tissue, while the physical properties of ice make it appear as a signal void in most conventional MRI sequences, as shown in Figure 2.

All of this, coupled with MRI’s ability to acquire volumetric, 3D images, makes it the most effective imaging modality for determining the extent of an ice ball during ablation. Though it is not often thought of as a real-time imaging modality, MRI sequences with acquisition times rapid enough to support near real-time intraoperative imaging and monitoring are used to observe ice ball growth and shrinkage during the freeze and thaw cycles of the ablation process [30,43]. The 0 °C degree isotherm is easily visualized on MRI but does require estimation of the −20 °C degree lethal isotherm just within the visualized iceball [42,44]. Furthermore, advanced MRI techniques like MR thermometry [45] may be used during the ablation to quantitatively track changes in temperatures, thereby providing even greater assurance that the targeted tissues have been sufficiently ablated.

Conversely, MRI comes with several practical limitations due to its eponymous high-strength magnetic fields. All supplemental equipment and hardware must be MR-compatible, built of materials that will not be subject to forces exerted by the magnetic fields. Even nonferrous, MR-compatible equipment, such as titanium cryoprobes, may still cause artifacts in MR images by distorting the local magnetic fields, which can make it challenging to accurately determine the location of the cryoprobes within the prostate prior to initiating the freezing cycle. Finally, MR is simply an expensive modality, which introduces additional pressure on clinical services that rely on it, further encouraging fast and efficient procedures.

Computed tomography offers high-resolution volumetric imaging akin to MRI, without the extensive technical limitations of working with high-strength magnetic fields. The soft-tissue contrast of CT is inferior to MRI, but generally sufficient to perform image-guided cryoablation [15]. Similarly, the iceball itself is visually distinct in CT, appearing as a well-defined hypodense region. Like MRI, the presence of cryoprobes can cause image artifacts: streaking due to denser metal of the probes relative to tissue [15]. Unlike ultrasound, CT does not generally support real-time imaging, making its intraoperative utility reliant instead on intermittent imaging. Another disadvantage of CT, relative to ultrasound and MRI, is its reliance on ionizing radiation to produce images. Though radiation exposure from medical imaging is extensively studied and generally considered an acceptable risk, it is best practice to minimize it as much as possible by making scans few and brief. A qualitative summary of the advantages and disadvantages of each imaging modality is collated in Table 1.

## 4. Narrative Literature Review

### 4.1. Search and Collection

As part of a supplemental narrative literature review, articles were sourced using several online databases and tools for locating academic literature (PubMed, Google Scholar, Scopus, Consensus). The search string “(prostate cancer) AND (cryoablation) AND (oncologic outcomes)” was used in PubMed, Google Scholar, and Scopus, with results restricted to only articles published in 2019 or after. For Consensus, a novel, large language model-based tool, this same query was phrased in natural language to perform a search. Inclusion criteria required, at a minimum, oncologic outcomes at or after 1 year post-treatment for patient cohorts of 50 or more. Other reviews were excluded. Studies with partially overlapping cohorts were not excluded. Article search and collection were performed by a single reviewer in March and April of 2025. To better represent differing approaches within the cryoablation space, specific effort was given to locating articles that reported salvage cryoablation, as a majority of the located articles were concerned with primary treatments. As this is a narrative review rather than a systematic one, it is not intended for this selection of articles to be comprehensive; rather, it is intended to give an overview of the current scientific landscape within this domain. In total, a selection of 15 articles was collated for more detailed review. The information from each article, including lead author, publication year, timescale, patient number, relevant cancer risk groups and treatment types, as well as reporting metrics, is depicted in Table 2. Metrics for characterizing oncologic and functional outcomes, while not wholly uniform between studies, were aggregated within reasonable bounds of comparison and noted in Table 2. For oncologic outcomes, the timeline of biochemical recurrence (BCR) was treated as the primary biomarker. Phoenix criteria for BCR [46] were preferred, but in studies where this criterion was not used, the closest reported metric was logged instead. Often, this was clinically significant prostate cancer (csPCa) as determined by a follow-up biopsy revealing Gleason Group 2 or higher PCa, or treatment failure as defined by the prescription of a secondary treatment post-cryoablation. These values were reported at different timelines following treatment across the studies; values were collated for BCR rates at 1, 2, 3, and 5 years post treatment. Erectile dysfunction and urinary incontinence reported by each article were logged only as a binary: the presence or absence of the morbidity. Individual studies determined these morbidities at varying thresholds and timelines. Where applicable, the morbidity rates reported at the longest timepoint posttreatment were used.

Considering the broad and rapidly growing nature of the topic, the intent is to provide an overview of the current research and literature landscape, providing resources to those who may wish to investigate further. The purpose of this article is to discuss the efficacy and safety of cryoablation by providing an overview of recent work in the domain. Comparable outcomes are reviewed for more traditional therapies to provide greater context for the role of cryoablation. The primary mechanisms for efficacy and safety assessments were biochemical recurrence-free survival and reports of post-treatment morbidities, respectively.

### 4.2. Literature Review

The reviewed literature consisted of a collection of 15 manuscripts reporting cryoablation outcomes, published in 2019 or later. Patient counts ranged from 50 to 260, and short-to-mid-term oncologic outcomes were reported on timelines between 1 and 10 years. As only two publications, Chen et al. (2023) and Tan et al. (2022), reported timepoints beyond 5 years, data were only logged up to that point [50,52]. Most studies were weighted towards shorter timelines since cryoablation is still an emerging technique. Patient selection criteria for cryoablation are often stratified by cancer risk groups and sorted into low-, favorable or unfavorable intermediate-, or high-risk, based on tissue Gleason Score. A majority of studies included patients from all risk groups, while the remainder largely considered some combination of intermediate- and high-risk cancers. Some studies sought to compare long-term outcomes between differing risk groups with the hypothesis that lower-risk cancers would correspond to better oncologic outcomes. Among those that performed this analysis, the results were mixed [34,48,49,55,56]. 

Oncologic outcomes were predominantly reported using biochemical recurrence-free survival or failure-free survival (FFS) at one or more timepoints post-treatment. In studies where both were reported, only BCRFS was recorded for consideration here. Biochemical recurrence, for BCRFS, was determined via the Phoenix criteria: PSA levels rising by +2 ng/mL from their post-treatment nadir [46]. Treatment failure, for FFS, was often characterized by the presence of Gleason Grade 2 or higher cancer in a post-treatment biopsy. Due to the heterogeneity of reported data, in-depth mathematical analysis of these rates is not supported. As a survival metric, these values decrease over time in studies that are reported at multiple longitudinal timepoints. Though some variation between salvage versus primary and whole versus partial gland ablation is expected, insufficient data points were present to draw any meaningful conclusions.

Many publications reported secondary functional outcomes, including erectile dysfunction or urinary incontinence. Reporting metrics varied between publications; to provide a uniform metric here, simply the presence or absence of an ED or UI morbidity within a percentage of the study population was logged. In cases where ED or UI rates were given at multiple timepoints, only the value at the final timepoint was collated here. More detailed summaries of functional outcomes cannot be aggregated meaningfully in this overview, as there is not a standardized set of metrics reported in the reviewed literature.

Though the aim is to provide a narrative overview of prostate cancer cryoablation in the greater context of other treatment paradigms, the selection of publications referenced in this work is neither exhaustive nor unbiased. As the article selection process was performed by a single researcher, there remains the potential for bias within the article selection process. Additionally, the existing body of work, particularly outcome-reporting studies, is relatively small. Outcome reporting metrics also varied between publications. This limits the quality of potential systematic reviews in this body of work. Though BCRFS was used as an oncologic outcome metric by many, others opted to use FFS to characterize a cancer recurrence instead. These metrics, though similar in intent, are not equivalent. Several standardized tools exist for classifying erectile function and urinary incontinence, like the International Index of Erectile Function (IIEF) or Sexual Health Inventory for Men (SHIM) Score and International Prostate Symptom Score (I-PSS), but the timeline post-intervention can impact rates over time [60,61]. Long-term, prospective trials with standardized outcome measurements are needed.

## 5. Other Prostate Cancer Treatments

Modern advances and an increased focus on minimizing morbidity risk have allowed cryoablation to occupy a growing portion of the treatment space, but more traditional approaches to PCa therapy remain dominant. Depending on the cancer staging and/or recurrence status, it can be prudent to treat prostate cancer using more than one approach [62].

The most traditional method of treating localized prostate cancer is the surgical removal of malignant tissues. Radical prostatectomy (RP), a complete surgical removal of the prostate and seminal vesicles, can be considered the gold standard of curative prostate treatments. Though very effective at removing malignant tissue, RP is associated with high rates of UI and ED morbidities, in addition to the standard set of risks associated with invasive surgical procedures. Though the traditional approach in an open RP operates via a single large incision, less invasive laparoscopic or robotic approaches are performed with the intention of lessening morbidity risk [9]. Depending on the location of the cancer and the preference of the patient, additional care may be given to spare the local nerves responsible for erectile function [6]. Surgery may also be performed with or without pelvic lymph node dissection. Patients with unfavorable intermediate and high-risk disease and/or concerning pre-surgical imaging findings are more likely to receive pelvic lymphadenectomy as guided by several nomograms, like the Briganti and MSKCC nomograms. Additional risks of lymphadenectomy include lymphocele, lymphedema, vascular or nerve injury, longer operative time, and recovery [63]. Surgical resection, depending on cancer risk and approach taken, can have biochemical recurrence-free survival rates generally between 60 and 80% with decreasing rates over time and with risk classification [64,65].

Prostate cancers may instead be treated noninvasively using external beam radiotherapy (EBRT) as an alternative to surgery. A beam of radiation is generated by a linear accelerator and fired at the prostate from multiple angles, inducing cell death preferentially in regions with overlapping radiation, leading to a high exposure. This approach is not associated with long recovery times, though treatments are typically fractionated over several weeks to months, requiring a series of visits to the medical service provider for treatment [66]. EBRT is associated with ED, UI and urinary retention in patients with benign prostatic hyperplasia, though the severity varies depending on the radiation delivery plan and the ability to avoid local nerves and organs [66]. Further, as a treatment modality utilizing ionizing radiation, there are several radiation-specific risks incurred by EBRT, such as gastrointestinal or genitourinary toxicity or the potential for secondary malignancies [66,67,68]. Radiotherapy is generally effective at managing oncologic outcomes in PCa, with 5-year BCRFS rates being reported at 71.7–82.6% in recent publications [65,69].

Another radiation-based treatment modality, brachytherapy, can deliver radiation to malignant tissues far more locally, without the skin dose risks of EBRT. In brachytherapy, small radiation sources are implanted into the prostate using minimally invasive surgical techniques. In place, each source emits radiation directly into adjacent tissues, treating nearby cancers without exposing more distal tissues to undue radiation. Sources may be temporary high-dose-rate (HDR) seeds that are implanted for a brief time, then removed, or low-dose-rate (LDR) seeds that are simply left in and permitted to decay to background levels of radioactivity [70]. For focal brachytherapy treatments, 5-year biochemical recurrence rates are approximately 9–18% [71,72]. Brachytherapy bears a similar risk profile to EBRT in terms of morbidity, albeit with a relatively higher risk of genitourinary morbidities and lower risk of bowel toxicity [73].

Other than cryoablation, there are several heat-based ablation methods for tissue destruction in the prostate. High-intensity focused ultrasound uses ultrasound energy, directed from transrectal or transurethral transducers, to generate heat at targeted locations in the prostate for focal or whole gland ablations [74,75]. Laser Fiber Ablation is another focal heat-based ablation technique, though it requires minimally invasive placement of laser fibers into the tissue, akin to the placement of cryoprobes in cryoablation [76]. All of these techniques ablate tissue by inducing heating and are prone to the same set of UI and ED morbidities as previously mentioned, as the thermal energy propagates through the tissues. This risk is mitigated with careful planning of the treatment zone margins and, if available, thermometry techniques to track the distribution of heat throughout the tissue, to spare critical structures [77]. Thermal ablation can vary in efficacy, with techniques like HIFU and laser ablation reporting BCRFS rates ranging between 45.2 and 85.7%, varying by cancer risk group [78,79].

Finally, there are a few methods of nonthermal ablation that can be used to treat prostate cancer, though they remain less common or restricted to specific use cases. Photodynamic Therapy (PDT) primes the patient by intravenously infusing a photosensitizing agent, which, when subject to light induced by a transperineally inserted probe, leads to brief, short-range chemical reactions that destroy local tissues [80]. Irreversible electroporation (IRE) uses needle-like electrodes inserted into the prostate to induce an electric current that disrupts the cellular membranes of local tissues, causing cell death with little to no thermal effects and preserving local noncellular structures [81]. Recent studies on mid-term outcomes from PDT and IRE have shown biochemical recurrence-free survival on the order of 50% at 3.5 years and 61% at 3 years, respectively [82,83], though more long-term studies are needed for meaningful comparison.

## 6. Conclusions

Cryoablation is an effective and well-tolerated treatment for prostate cancer with comparable oncologic and functional outcomes in early studies. While additional high-quality data with standardized endpoints and consistent metrics are needed before incorporation into current treatment algorithms, the role of cryoablation is continuing to develop with a heavier emphasis on salvage treatment for recurrent disease. Treatment of prostate cancer recurrences, either within the gland or in the pelvis, remains a key advantage of cryoablation, as additional surgery or radiation may be contraindicated. When compared against more traditional approaches, focal cryoablation is considered to be advantageous for patients who wish to minimize the risks of morbidities commonly associated with PCa treatment or for those who are not candidates for other standard of care therapies. Advanced imaging techniques have provided a more reasonable backdrop for focal therapy, improving detection and diagnosis, treatment accuracy, and post-ablation monitoring. Ultimately, the optimal approach for prostate cancer treatment is multi-disciplinary and determined by many factors, including severity, staging, available treatment paradigms/recommendations, patient-specific factors, and patient preferences. Cryoablation offers an additional treatment modality to this well-defined space to provide an additional safe and effective treatment option to patients and clinicians.

## Figures and Tables

**Table 1:** Imaging modality data is summarized for ultrasound, MRI, and CT applications in cryoablation. In general, ultrasound can be thought of as the most accessible, but lowest in image quality. MRI occupies the far end of the spectrum, with CT in the middle. Procedural limitations refer to constraints on clinicians during the procedure, such as the limited space of operating within a bore or the high magnetic fields present in MRI.

Modality	Availability	Cost	Time Resolution	Soft Tissue Contrast	Ice Ball Clarity	Radiation Exposure	Procedural Limitations
Ultrasound	High	Low	High	Medium	Moderate	None	Low
MRI	Low	High	Medium	High	High	None	High
CT	Moderate	Moderate	Low	Low	Low	Moderate	Medium

**Table 2:** Across 15 publications from the past 6 years, timelines, patient demographics, procedure types, and patient outcomes were collated [34,38,47,48,49,50,51,52,53,54,55,56,57,58,59]. Biochemical recurrence-free survival (BCRFS) or the closest reported analog was collected for 1-, 2-, 3-, and 5-year periods after treatment. Most logged values were reported directly within the named publication, though some were derived from tables or other supplemental materials.

Author	Year	Timeline	N	Risk Group(s)	Type	Scope	Report Metric	1 yr	2 yr	3 yr	5 yr	ED	UI
Shah et al.	2019	3 yr	122	Intermediate, High	Primary	Focal	FFS: Second Treatment			83.2%		16.1%	0.0%
Oishi et al.	2019	5 yr	160	Intermediate, High	Primary	Focal	BCRFS: Phoenix				62.0%	27.0%	3.0%
Oishi et al.	2019	5 yr	94	All	Primary	Whole	BCRFS: Phoenix				81.0%	89.0%	4.0%
Tan et al.	2022	9 yr	260	All	Primary	Whole	BCRFS: Phoenix	97.0%		92.0%	89.0%		3.0%
Baskin et al.	2022	5 yr	75	Intermediate, High	Primary	Focal	BCRFS: Phoenix				69.4%		
Chen et al.	2023	10 yr	191	High	Primary	Whole	BCRFS: Phoenix	92.6%		76.6%	66.7%	88.9%	2.6%
Tan et al.	2023	5 yr	110	All	Salvage	Whole	BCRFS: Phoenix	85.0%	81.0%	79.0%	71.0%		9.0%
Campbell et al.	2023	5 yr	63	All	Salvage	Focal	BCRFS: Phoenix		79.0%		60.0%	25.6%	4.9%
			63						80.0%		70.0%	14.1%	16.0%
Wysock et al.	2023	3 yr	132	Intermediate	Primary	Focal	FFS: Biopsy			86.0%		7.0%	0.0%
Sevlaggio et al.	2023	5 yr	110	All	Primary	Focal	BCRFS: Phoenix				68.5%		6.3%
			27	Low							78.8%		
Khan et al.	2023	5 yr	115	Intermediate	Primary	Focal	BCRFS: Phoenix				74.0%	3.1%	1.8%
			23	High							55.0%		
Zhu et al.	2023	2 yr	75	Intermediate, High	Primary	Focal	FFS: Biopsy	66.0%	57.0%			24.0%	0.0%
Vieira e Brito et al.	2023	1 yr	55	All	Salvage	Whole	BCRFS: Phoenix	85.0%					38.2%
Marquis et al.	2025	2 yr	50	Low, Intermediate	Primary	Focal	FFS: Biopsy		85.6%			15.0%	0.0%
Mate et al.	2026	3 yr	111	All	Primary	Focal	FFS: Biopsy			63.0%			

## Data Availability

No new data were created or analyzed in this study.

## Abbreviations

The following abbreviations are used in this manuscript:

PCa	Prostate Cancer
BPH	Benign Prostate Hyperplasia
CT	Computed Tomography
AUA	American Urological Association
PSA	Prostate-Specific Antigen
GS	Gleason Score
ADT	Androgen Deprivation Therapy
AE	Adverse Event
ED	Erectile Dysfunction
UI	Urinary Incontinence
OAR	Organ at Risk
PET	Positron Emission Tomography
TRUS	Transrectal Ultrasound
BCR	Biochemical Recurrence
csPCa	Clinically Significant Prostate Cancer
BCRFS	Biochemical Recurrence-Free Survival
FFS	Failure-Free Survival
IIEF	International Index of Erectile Function
SHIM	Sexual Health Inventory for Men
RP	Radical Prostatectomy
EBRT	External Beam Radiotherapy
HDR	High Dose Rate (Brachytherapy)
LDR	Low Dose Rate (Brachytherapy)
PDT	Photodynamic Therapy
IRE	Irreversible Electroporation
